# Modeling heart rate of individual and team manual handling with one hand using generalized additive mixed models

**DOI:** 10.1186/s12874-024-02169-7

**Published:** 2024-02-15

**Authors:** Mohammad Hamed Hosseini, Rashid Heidarimoghaddam, Mehrdad Anbarian, Saeed Ilbeigi, Leili Tapak

**Affiliations:** 1 Department of Ergonomics, School of Public Health, Research Centre for Health Sciences Hamadan University of Medical Sciences, Hamadan, Iran; 2 Department of Sports Biomechanics, Faculty of Sport Sciences, Bu-Ali Sina University, Hamadan, Iran; 3 Sports Biomechanics, Faculty of Sport Sciences, University of Birjand, Birjand, Iran; 4 Department of Biostatistics, School of Public Health and Modeling of Noncommunicable Diseases Research Center, Hamadan University of Medical Sciences, Hamadan, Iran

**Keywords:** Team manual handling, Heart Rate, Generalized Additive Mixed Model

## Abstract

**Objectives:**

Despite the fact that team manual handling is common in different working environments, the previous studies in this regard, particularly those with a physiological approach are quite limited. The present study is an attempt to model the heart rate (HR) of individual and team manual handling with one hand*.*

**Methods:**

Twenty-five young men (aged 21.24±1.42 year) volunteered for this study. The experiments included individual and two-person handling of the load with three different weights with and without height difference. The participants’ HR was registered at the end of the route by a chest-strap pulse monitor and a polar watch according to the manufacturer's recommendation*.* A multivariate Generalized Additive Mixed Model (MGAMM) was used for modeling heart rate based on explanatory variables of workload, carry method, HR_*rest*_, body weight, height, knee height, shoulder height, elbow height, and hand height*.* The significance level of the tests was considered as <0.05.

**Results:**

Based on the MGAMM, the average HR (bpm) of participants increased as the workload increased (*P*<0.001). Handling the load with a taller person increased the HR compared to shorter partner (*P*<0.001). Moreover, the nonlinear associations of the resting HR (*P*<0.001), body weight (*P*<0.001), height (*P*<0.001), and the height of elbow, hand and knee (*P*<0.001) were statistically significant. The adjusted R^2^ of the model was 0.89 indicating that about 90 percent of the variations observed in HR could be explained by the variables in the model. This was greater than the model considering only linear effects (*R*^2^ =0.60).

**Conclusion:**

The model obtained in this study can predict the heart rate of individual and team one-handed handling with high validity. The MGAMM can be used in modeling heart rate in manual handling.

## Introduction

Manual materials handling is one of the main causes of fatigue and reduced performance in many workplaces. Researchers have always sought appropriate strategies to mitigate these consequences [1–3]. In terms of the number of people involved in handling a load, MMH can be individual or team-based. In various industrial, military, construction, medical, and agricultural environments, when the weight of the load exceeds the capacity of an individual or is large in size, and it is not feasible for the person to lift or carry the load using mechanical equipment, the team steps in to move the load [4–8]. Team manual handling is a method of manual handling in which the materials are moved through tasks such as lifting, carrying, lowering, pushing, and pulling by two or more persons.

Two-person handling is one of the common methods of team manual handling that can be performed with one or two hand (s). When using two hands in tandem or parallel, each method has its specific a particular shortcoming. In tandem, where two workers carry a load with two hands, they cannot see obstacles on the path, posing a high safety risk of falling due to the inability to detect obstacles. Additionally, based on previous findings, two-person handling in parallel is considered difficult by workers [3]. Consequently, team members may choose to carry the load with one hand on the unilateral side of their body.

Based on the current knowledge, despite team manual handling being common in various working environments, studies in this regard, especially those with a physiological approach, are limited [9]. The few studies in this area have been focused on measuring heart rate (HR) in team manual handling using two hands [3, 10, 11]. For instance, Wu and Chung examined the effects of carrying methods (parallel and tandem) with and without box handles on heart rate during a two-person carrying task (two-hands setting), involving 16 female participants [3]. Additionally, Visser et al conducted an observational study evaluating team lifting on work demands and workload (measured by as the percentage of heart rate reserve) among ironworkers [11]. Based on the results of a recent systematic review study by the authors of this study [9], there are no studies examining the physiological parameters of the body in team manual handling with one hand. Furthermore, the impact of variables such as load weight, body weight, team members’ height difference, and body dimensions on these parameters has not been explored. Previous studies have indicated that these variables affect physiological parameters in individual manual handling [12–16]. It is anticipated that these variables will similarly influence the physiological parameters of the body in team manual handling with one hand. Therefore, this study was conducted with the aim of investigating these influences and modeling the heart rate during both individual and team manual handling with one hand. To achieve this, we employed the multivariate generalized additive mixed model (MGAMM) as a state-of-the-art and advanced statistical model to capture the correct functional form of the variables in the model.

## Materials and methods

### Study subjects

Twenty-five men volunteered for this study. The experiments were conducted based on the protocol approved by the ethics committee of the university. Before the experiments, the participants were briefed on the study's purpose, and written consent was obtained from them. The inclusion criteria comprised men with an age range of 20-25 years who volunteered to participate, while the exclusion criteria encompassed a history of drug use, smoking, accidents, surgery, or respiratory, musculoskeletal, and cardiovascular diseases.

### Experiment procedure

The participants received the required instructions 72 hours before the load carriage experiments. They were instructed to have their breakfast at least two hours before the experiment and were asked not to engage in heavy exercises one day before. Participants wore light cotton clothes and sports shoes. Before each experiment, their resting heart rate (HR_rest_) was measured.

The load carriage experiments involved individual and two-person handling of loads with three different weights, using the dominant hand on the unilateral side of the body. This process included lifting from knuckle height, carrying in a 30-meter route with a slope of 0° and a step rate of 100 steps/min, and lowering at knuckle height. The step rate was regulated by a metronome. Walking time in each experiment was measured with a chronometer. If any unwanted delay occurred during the route, such as stopping or not syncing with metronome beat, the experiment was repeated. In individual handling, the load weights were 3.5, 7, and 10.5 kg. The highest load weight was determined based on the findings of previous studies [17–19]. The light and heavy weights were selected with proportional weight intervals.

Two-person handling also involved managing loads with three different weights: 7, 14, and 21 kg (twice the weight used in individual handling), following the same route with the mentioned features. As a result, each person's share of the weight during team handling equaled their share in individual handling. In this stage, evaluation was conducted for only one of the team members.

For a more thorough examination of the impact of the team members’ height difference on their heart rate, three individuals with approximately the same height, about 12 cm taller, and about 12 cm shorter than the person under examination were included to assist in handling the load. Consequently, the number of experiments in the team handling stage was 9.

The sequence for all the mentioned experiments was selected randomly. Between every two experiments, each participant rested sitting for 10 minutes to allow their heart rate to return to resting levels (HR_rest_). All experiments took place in a controlled laboratory setting with a temperature range of 20-23°C and a relative humidity of 35-40%. The temperature and humidity were regulated by an air conditioning system.

The load used for individual handling included a box with dimensions of 40×16×30 cm (length × width × height) and a handle diameter of 3 cm. The load used for two-person handling was a box with dimensions of 70×40×30 cm and handles diameter of 3 cm. These boxes were designed to resemble industrial boxes. The weights inside the boxes were arranged to ensure equal distribution throughout the box. Participants' heart rate was recorded at the end of the route. To measure their HR during the experiments, a chest-strap pulse monitor and a watch (A300; Polar Electro Oy; Finland) were utilized.

### Statistical analyses

A repeated measurement test was employed to compare the average heart rate between different handling methods. A graph with error bars was used to illustrate the variables. A generalized additive mixed model was utilized for modeling heart rate. Let ${y}_{im}$ denote the heart rate of the $i$ th individual ($i=\mathrm{1,2},\dots ,n$ ) for the carry method ($m=\mathrm{1,2},\mathrm{3,4}$) and let ${x}_{i}^{T}=\left(1,{x}_{i1},\dots ,{x}_{ip}\right)$be the covariate vector associated with subject $i$ (here, including body weight, height, knee height, shoulder height, elbow height, and hand height). Also, let ${\eta }_{m}$be the random effect for the $i$ th individual (here each individual creates a cluster) and ${\varepsilon }_{im}$ be the random error term (normally distributed). To consider the nonlinear relationship between HR and the continuous variables in the model, a semi-parametric model including both parametric and non-parametric components was used as follows:

$$y_{im}=\alpha+\beta_{wl}\times workload+\beta_{HS}\times Handlig\_states+\sum_{k=1}^Kf\left(x_{ik}\right)+\eta_m+\varepsilon_{im}$$

where,$f\left({x}_{ik}\right)$ indicates a smooth function of anthropometric variables and resting heart rate, $\alpha$ is a constant term (intercept) and ${\beta }_{wl}$ and ${\beta }_{HS}$ are two vectors consisting of the dummy variables for workload (3.5 (Reference category)), 7 and 10.5 kg) and handling states levels (individual, same height, taller and shorter (Reference category)). In this study we used cubic spline method to estimate the functional form of $f\left({x}_{ik}\right)$ s. All the analyses were done on the R v.4.1.2. package “mgcv”and the differences were considered as statistically significant at *P*<0.05. The normality assumption was checked using Kolmogorov-Smirnov test. The null hypothesis was “the normality assumption holds”.

## Results

### Statistics

The mean and standard deviation related to the participants’ age were 21.24±1.42 years (age range 20–25 years), 67.16±9.34 kg for their weight, and 21.63±2.35 for their body mass index. According to their statements, all the subjects were right-handed. Anthropometric dimensions of participants are presented in Table 1.

**Table 1:** Anthropometric dimensions of the participants in cm

**Height**	**Shoulder height**	**Elbow height**	**Knuckle height**	**Knee height**
**175.96±5.99**	143.00±6.63	111.00±5.09	76.00±4.07	46.80±2.88

Based on the length of the load-carrying route and the duration of the walking, the participants’ walking speed in each experiment was calculated. The results showed no significant difference in the walking speed among the participants during the 12 experiments (*p* > 0.05). The mean score and standard deviation related to the participants’ walking speed were found to be 1.10±0.05 m/s.

### Comparison of the average heart rate in various settings

**Fig. 1:**
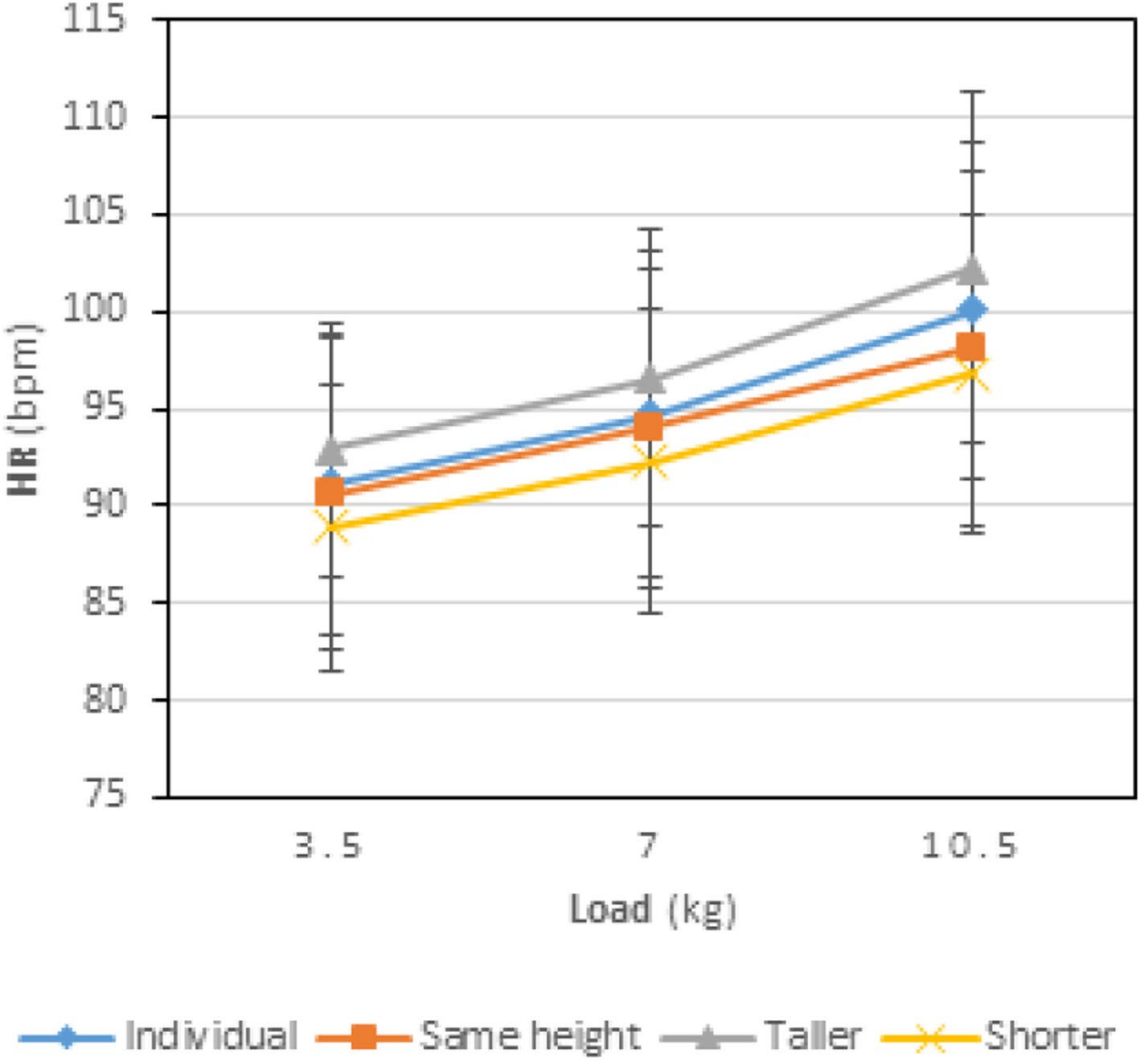
The effect of load weight and handling states on the participants’ HR (bpm)

The mean and standard deviation related to the participants’ resting heart rate (HR rest) was 69.96±4.63 bpm. Table 2 presents the participants’ heart rate in different states of individual and team handling with various weights. It should be noted that as each person’s share of the weight in two-person handling was equal to their share in individual handling, the load weights in the table and figure are the same as those in individual handling. In all states of handling, following an increase in the load weight, heart rate also increased; this increase was significant in all these states (*P* < 0.05). Furthermore, some differences were found between individual and team handling, especially when the team members were not of the same height. Some of these differences were significant. Figure 1 illustrates these differences.

**Table 2:** Comparing the mean related to participants’ heart rate (bpm) for different weights and states of handling

**Load kg**	**Handling states**				*P-value*
	Individual	Two-person			
		Same height	Taller	Shorter	
3.5	91.16±7.73^a^	90.56±7.99^a^	92.92±6.52^ab^	88.88±7.41^a^	<0.001
7	94.72±8.44^a^	94.04±8.20^a^	96.52±7.65^ab^	92.28±7.84^a^	<0.001
10.5	100.08±8.65^ab^	98.12±9.09^a^	102.20±9.04^abc^	96.84±8.20^a^	<0.001
*P-value*	**<0.001**	**<0.001**	**<0.001**	**<0.001**	<0.001

^a^ There is a significant difference between the three weights in the same state of handling (*p* < 0.05).

^b^ There is a significant difference with the shorter member in the same weight (*p* < 0.05).

^c^ There is a significant difference with the same-height state in the same weight (*p* < 0.001).

### Multivariate generalized additive mixed model of heart rate

Table 3 shows the results of the multivariate generalized additive mixed model in modeling heart rate based on variables such as workload, carry method, resting heart rate (HR_rest_), body weight, height, knee height, shoulder height, elbow height, and hand height. The first part of the table illustrates the association between workload and handling states.

**Table 3:** The results of the Generalized Additive Mixed Model in modeling heart rate

**Parametric coefficients**	**β**	**Standard Error**	**Test statistics**	***P*-value**
**Intercept**	85.214	1.0163	83.850	< 2e-16
**Workload**				
3.5 kg (Reference category)				
7 Kg	3.492	0.413	8.560	1.73E-15
10.5 Kg	8.412	0.413	20.376	< 2e-16
**Handling states**				
1. shorter (Reference category)				
2. same height	1.599	0.478	3.347	0.000932
3. individual	2.655	0.477	5.568	6.18E-08
4. taller	4.547	0.493	9.540	< 2e-16
**Approximate significance of smooth terms**	Edf	Ref.df	F	*P*-value
S(HR_rest_)	3.184	3.586	87.837	< 2e-16
S(body weight)	2.560	2.869	4.675	0.00595
S(height)	3.165	3.328	4.480	0.00393
S(knee height)	2.507	2.700	14.395	5.97E-07
S(shoulder height)	1.000	1.000	44.313	< 2e-16
S(elbow height)	4.419	4.568	45.182	< 2e-16
S(hand height)	4.822	4.900	18.176	< 2e-16

As observed, the associations were highly significant, such that an increase in workload from 3.5 to 7 kg was related to an average heart rate increase of about 3.5. Additionally, increasing the workload from 3.5 to 10.5 kg was associated with an average heart rate increase of about 8.5.

**Fig. 2:**
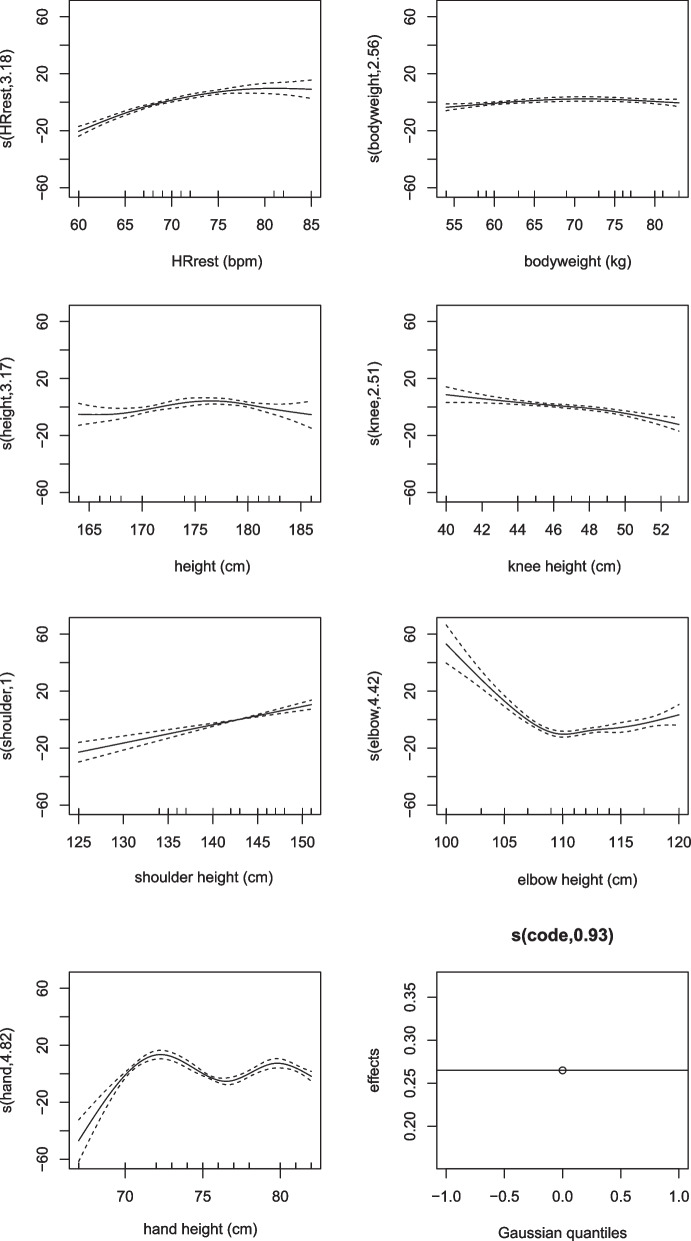
The relationship between HR_rest_, body weight and anthropometric dimensions with heart rate. Note: The numbers of the vertical axis indicate the estimated degree of freedom

Moreover, the carry method was significantly associated with an increase in heart rate. For instance, the heart rate increased by 4.5 for taller members compared to shorter members on average, when other variables were held constant. The lower part of Table 3 indicates the *p*-values for the nonlinear effects. As shown, all variables were nonlinearly associated with heart rate. The functional forms of the relationships are depicted in Fig. 2

The adjusted R^2^ was 0.889, indicating that about 90 percent of the variations observed in heart rate could be explained by the variables in the model, which was greater than the model with a linear assumption of the relationships (R^2^ was about 0.6).

**Fig. 3:**
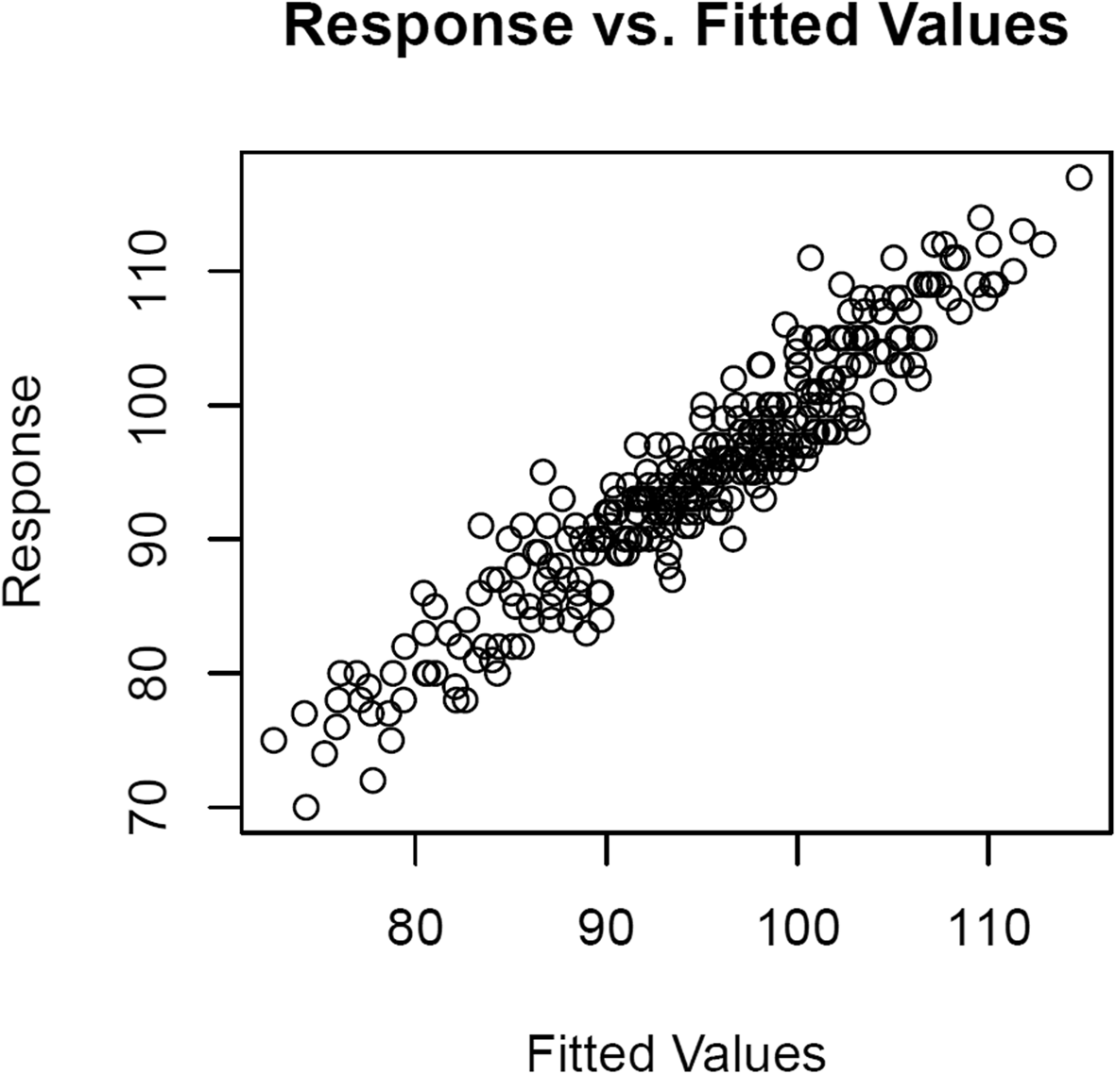
Observed response vs. fitted values using GAMM

The goodness of fit of the model was visually checked using the “mgcv” package. The results are shown in Fig. 3 According to the plots, there was a strong association between observed heart rate (HR) and fitted values using GAMM. Also, no serious violations of the normality of residuals were observed (results not shown). We also used the Kolmogorov-Smirnov test to check the normality of the residuals. The results showed that there was no evidence of a violation of normality for the residuals (*p* > 0.05)

## Discussion

The present study aimed to examine the effect of load weight, handling method, team members’ height difference, and body dimensions on heart rate in both individual and team one-handed handling. The analysis of the effect of load weight on the participants' heart rate indicated that as the load weight increased in all methods of individual and team handling, the participants' heart rate also increased. Previous studies on individual handling have also reported a significant effect of load weight on increasing heart rate [20–23].

In the current study, the effect of the participants' height difference on the heart rate of team members during carrying was also examined. The results showed that the height difference between team members can have a significant effect on these responses. When the height of the participant in team handling is greater than that of the person assisting with handling the load, their mean HR was higher, and when their height is shorter, the mean score related to these responses is lower. These differences were significant in all three load weights (*P* <0.05). Compared to the same-height condition, when the participant’s height was higher, a significant difference was observed in HR in the third load weight (*P* <0.001).

The findings of the present study suggest that in two-person handling, when there is a height difference between team members, the taller person physiologically experiences more stress and is at a higher risk of injury. There are no similar studies on the effect of the height differences of team members on their physiological parameters

In the present study, for a more careful examination of the effect of individual and team handling on the participants' heart rate, the load weight for two-person handling was two times higher than in individual handling. Therefore, each person’s share of the load weight in team handling was equal to their share in individual handling. Based on the findings, when there was no height difference between team members, the mean scores related to heart rate were different across the three weight conditions; these responses were slightly smaller in the team handling state compared to individual handling, but none of these differences was significant. In the study by Wu et al. [3]. in which there was no considerable difference in the team members’ height, the difference in heart rate between individual and team handling was not significant.

In previous studies, the maximum weight recommended for continuous handling tasks with one hand for men has been 10 kg to ensure that heart rate (HR) does not exceed 100 bpm [17–19, 24]. The results of the present study showed that the mean HR of the participants did not exceed the recommended value, except for taller members in team handling with the third load weight, where the obtained result was slightly higher than the recommended limit.

The model presented in this article has high validity and demonstrates non-linear relationships between heart rate and the investigated variables. The presented diagrams illustrate the details of these non-linear relationships. Some relationships closely resemble a linear pattern, while others are entirely non-linear. Some previous studies assumed a linear relationship between heart rate and variables such as anthropometric parameters, providing a predictive model based on the covariates in the model [25, 26]. Ismaila et al showed that there is a non-linear relationship between HR at work and demographic variables [27]. The connections between subjects’ characteristics and HR may be non-linear. Considering a linear relationship may lead to biased estimates of the true effects of variables in the model and, consequently, provide erroneous predictions. The results of the present study are more similar to the results of Ismaila et al.

## Strengths and Limitations

In the current study, for a more careful examination of the effect of load weight and handling method on heart rate (HR), an attempt was made to maintain a fixed walking speed as much as possible. This was achieved by using a metronome that regulated a consistent stepping rate during the 12 experiments. The decision to use a metronome aimed to ensure a fixed walking speed, as previous studies using a treadmill have been associated with overestimation of physiological responses [28, 29]. Employing this method was an effort to avoid the occurrence of this error, which represented a strength compared to previous studies.

This study specifically focused on heart rate during two-person handling in a laboratory setting with young participants. Future studies could further investigate these responses in larger teams, incorporating members from different age groups, within a real workplace.

## Conclusions

So far, the few studies focused on team handling have been limited to team handling with two hands, and team handling with one hand has not been addressed. The present study focused on this method of manual handling. The model obtained in this study for the prediction of HR while manual handling can predict the heart rate of individual and team one-handed handling with high validity. The Generalized Additive Mixed Model can be used in modeling heart rate in manual materials handling.

## Data Availability

The data are available upon reasonable request from the corresponding author.
